# Role of SP-1 in SDS-Induced Adipose Differentiation Related Protein Synthesis in Human Keratinocytes

**Published:** 2007-10-04

**Authors:** Emanuela Corsini, Omar Zancanella, Laura Lucchi, Barbara Viviani, Marina Marinovich, Corrado L. Galli

**Affiliations:** Laboratory of Toxicology, Department of Pharmacological Sciences, University of Milan, Via Balzaretti 9, 20133 Milan, Italy

**Keywords:** skin irritation, transcription factors, mithramycin, calcium

## Abstract

Skin irritation is a complex phenomenon, and keratinocytes play an important role in it. We have recently characterized the expression and protective role of adipose differentiation related protein (ADRP) in skin irritation. In particular, ADRP expression is induced to recover functional cell membrane following the cell damage caused by skin irritants.

The purpose of this study was to characterize in a human keratinocyte cells line (NCTC 2544) the biochemical events that lead to ADRP expression following SDS treatment, and in particular, to investigate the role of transcription factor SP-1. Analysis of ADRP promoter region revealed the presence of a potential binding site for the transcription factor SP-1 close to the start site. Evaluated by measuring the DNA binding activity, we found that SDS induced a dose and time related SP-1 activation, which was correlated with SDS-induced ADRP mRNA expression. Furthermore, SDS-induced SP-1 activation, ADRP mRNA expression and lipid droplets accumulation could be modulated by mithramycin A, an antibiotic that selectively binds to the GC box preventing SP-1 binding and gene expression. This demonstrated that SDS-induced ADRP expression was mediated in part through the transcription factor SP-1. In addition, SDS-induced SP-1 activation and ADRP expression could be modulated by the calcium chelator BAPTA, indicating a role of calcium in ADRP-induction.

Thus, every time an irritant perturbs the membrane barrier, it renders the membrane leaky and allows extracellular calcium to enter the cells, an event that provides the upstream mechanisms initiating the signaling cascade that triggers the activation of SP-1 and culminates in the enhancement of ADRP expression, which helps to restore the normal homeostasis and ultimately repairs the to membrane.

## Introduction

Skin irritation is, from both a clinical and a mechanistic perspective, a multifaceted disease. The biochemical mechanisms activated in contact dermatitis are complex and not fully understood, normally involving resident epidermal cells and fibroblasts of dermis, as well as, invading leukocytes, interacting with each other under the control of a network of cytokines and lipid mediators (Boss and Kapsenberg, 1993; Corsini and Galli, 1998; 2000). In the last two decades, it has become clear that keratinocytes, which represent 95% of epidermal cells, play an important role in initiating and maintaining skin inflammatory and immunological reactions. Using genomic techniques, we have identified in human keratinocytes the upregulation of adipose differentiation related protein (ADRP) by skin irritants (Corsini et al. 2002). ADRP, the human homolog has been termed adipophilin, is a 53 kDa protein initially cloned from a differentiated murine adipocyte cDNA library (Jiang and Serrero, 1992). ADRP is a lipid droplet protein found in most cells and tissues, which has an important role in management of neutral lipid stores, both in deposition and mobilization (Heid et al. 1998). Intracellular neutral lipid storage droplets are essential cytosolic organelles of eukaryotic cells used mainly as energy source and for membrane biogenesis (Brasaemle et al. 1997; Miura et al. 2002). Lipid droplets provide fatty acids for signaling/gene regulation, fatty acids and glycerides for membrane phospholipid synthesis, as well as, fatty acids to produce energy (Brown, 2001).

Following ultrastructural examination, both in physiological and pathological conditions, the presence of neutral lipid droplets in keratinocytes has been occasionally described (Johnson et al. 1987; Kanerva, 1990; El-Shoura and Tallab, 1997). In physiological conditions, lipids are synthesized by keratinocytes and exported to the intercellular space to form the skin permeability barrier. On the contrary, the presence of numerous lipid droplets in the horny cells is a marker of abnormal keratinization and/or of a hyperproliferative effect.

Exposure of normal skin to organic solvents or detergents removes lipids affecting the barrier integrity, often induces irritant contact dermatitis (Imokawa et al. 1989; Grubauer et al. 1989). The disruption of the barrier function by lipid extraction is followed by a burst of epidermal lipid synthesis, including unsaponifiable lipids, fatty acids and sphingolipids to restore their normal level and barrier functions (Menon et al. 1985; Grubauer et al 1989; Holleran et al. 1991).

We have previously demonstrated the upregulation of ADRP in human keratinocytes by skin irritants, and we have also showed its protective role in SDS-induced cell damage (Corsini et al. 2003). ADRP expression was also induced in vivo in an experimental model of skin irritation, confirming the relevance of the in vitro model of irritancy (Corsini et al. 2003). We have proposed the following scenario: the surfactant effect of SDS damages lipid bilayers of cell membranes, rendering them leaky and allowing extracellular calcium entrance and activation of gene expression, including ADRP. Lipids deriving from damaged membrane are then reorganized into lipid droplets by ADRP and eventually reused to repair membrane. In parallel, disruption of the cell membrane leads to the release of proinflammatory cytokines, such as interleukin-1α, which in turn by acting in an autocrine manner may induce tumor necrosis factor-α, IL-8 and other inflammatory mediators release, resulting in skin irritation.

The analysis of 540 bp of 5′-flanking region of mouse ADRP gene for potential transcription factors binding sites revealed consensus sequences for SP-1, TRE, AP-2, NF-1, c-myc, Pax-2, two TRE half-sites, and a close match to EGR1 and WT1 (Eisinger and Serrero, 1993). However, little is known about the molecular mechanisms and promoter control elements, those regulate the transcription of the human ADRP gene. As in the mouse promoter region, the bioinformatic analysis of the 5′-flanking region of exon 1 of the human ADRP gene, revealed the presence of two potential binding sites for the transcription factor SP-1 close to the start site (in position −42 and −58). The purpose of this study was to conduct molecular mechanistic investigations and aimed to characterize the role of transcription factor SP-1 in SDS-induced ADRP expression in human keratinocytes. This is the first report of the role of SP-1 activation in the regulation of ADRP expression in human keratinocytes by skin irritants, offering insight into the mechanisms which serve to regulate ADRP transcription.

## Materials and Methods

### Chemicals

1,2-bis(2-aminophenoxyethane-N,N,N′, N′-tetraacetic acid acetoxymethyl ester (BAPTA), fura-2 pentakis(acetoxymethyl) ester (FURA 2-AM), nile red (9-diethylamino-5H-benzophenoxazine-5-one), 12-O-tetradecanoylphorbol 13-acetate (TPA) and sodium dodecyl sulfate (SDS) were obtained from Sigma (St Louis, MO, U.S.A.). Mithramycin was from Tocris (Bristol, U.K.). Antibodies against human ADRP FITC-conjugated were obtained from Research Diagnostic Inc. (Flanders, NJ, U.S.A.). Electrophoresis reagents were from Bio-Rad (Richmond, CA, U.S.A.). All other reagents, unless specified, were purchased from Sigma. All reagents were purchased at the highest purity available.

### Cells and experimental procedures

The human epidermal cell line NCTC 2544 (ICN Flow, Irvine, U.K.) was cultured in RPMI 1640 (Sigma) containing 2 mM L-glutamine, 0.1 mg/ml streptomycin, 100 IU/ml penicillin (media), and supplemented with 10% fetal calf serum (FCS, Sigma), at 37 °C in 5% CO_2_. For the majority of experiments, cells were cultured to confluence in 12 well plates. Confluent monolayers contained approximately 2 × 10^6^ cells. Then, cells were incubated for different times in the presence or absence of SDS or other compounds in 0.56 ml of media supplemented with 1% FCS, as indicated in the legend to figures.

To assess the involvement of SP-1, cells were treated for 1 h with the specific SP-1 inhibitor mithramycin or DMSO as vehicle control (final concentration 0.2%) in media supplemented with 1% FCS; then exposed to SDS for different times as indicated in the figure legends. To characterize the role of calcium, cells were incubated with the calcium chelator BAPTA (30 μM) for 30 min in media supplemented with 1% FCS. Monolayers were then washed once and cells were cultured with SDS for different time points.

### Transcription factor activation

Nuclear extract were prepared essentially as described by Schreiber et al. (1989). After treatment, cells were lysed in 0.4 ml of a hypotonic lysis buffer (10 mM Hepes, pH 7.8, 10 mM KCl, 2 mM MgCl_2_, 1 mM dithiothreitol, 0.1 mM EDTA, 0.1 mM phenylmethylsulfonyl fluoride). Cells were incubated on ice for 15 min and then 25 μl of a 10% Nonidet P-40 solution was added, and cells were mixed for 15 sec, and then centrifuged for 30 sec at 12,000 rpm. Pelleted nuclei were suspended in 50 μl of buffer C (50 mM Hepes, pH 7.8, 50 mM KCl, 300 mM NaCl, 10% glycerol, 1 mM dithiothreitol (DTT), 0.1 mM EDTA, 0.1 mM phenylmethylsulfonyl fluoride), mixed for 20 min, and centrifuged for 5 min at 12,000 rpm. The supernatants represented the nuclear extracts. Protein concentration was measured using a commercial kit (Bio-Rad). SP-1 activation was evaluated both by electrophoretic mobility shift assay (EMSA) as previously described (Corsini et al. 2001), and by a specific colorimetric method using a commercially available kit (BD Mercury TransFactor kit, BDBio-sciences, San Jose, CA, U.S.A.). Briefly, the TransFactor kit is a 96-well plate with oligonucloetides containing the consensus binding sequence for each transcription factors coated on the wells. When cell extracts containing the transcription factors are incubated in the wells, the transcription factors bind to their consensus sequences. Bound transcription factors are then detected by a specific primary antibody. A horseradish peroxidase-conjugated secondary antibody is then used to detect the bound primary antibody. The enzymatic product is then measured with standard microtiter plate reader. For EMSA, binding reaction mixtures (16 μl) containing 5 μg of protein of nuclear extract, 2 μg of poly (dI-dC)-poly(dI-dC), 10,000 cpm of ^32^P-labeled probe in binding buffer (10 mM Hepes, pH 7.9, 50 mM NaCl, 1 mM EDTA, 1 mM DTT, 10% glycerol, 1% Ficoll, and 0.2 mg/ml albumin) were incubated for 30 min at room temperature before separation in a 7% acrylamide gel in 1X TBE followed by autoradiography. A double-strand oligonucleodide containing the binding site for SP-1 (5′-ATTCGATATCGGGGCGGGGCGAGC-3′) - was labeled with α-^32^PdATP using T4 poly nucleotide kinase (Amersham, Buckinghamshire, U.K.).

### Fluorescence microscopy

Lipid droplets accumulation and ADRP expression were analyzed using, nile red and anti human ADRP antibody, respectively. Nile red is a vital stain for the detection of intracellular lipid droplets by fluorescence microscopy and flow cytometry (Greenspan et al. 1985). After treatment, monolayers were washed twice with PBS, cells were trypsinized, fixed in 2% formaldehyde for 20 min at room temperature, washed once with PBS, and stained with nile red in PBS for 10 min. Nile red was dissolved in ethanol at 1 mg/ml and then added to PBS to the final concentration of 10 μg/ml. After washing with PBS, cells were resuspended in 0.5 ml of PBS and a cytospin using 50 μl was done. For ADRP staining, cells were fixed and permeabilized after trypsinisation using Leucoperm^™^, following supplier’s instructions (Serotec, Oxford, U.K.). Cells were stained for 30 min at room temperature with 1:10 diluted fluorescein conjugated anti human ADRP. After washing with PBS, cells were resuspended in 0.5 ml of PBS and a cytospine of 50 μl was performed. In both cases, coverslips were mounted on glass slides using Permafluor (Lipshaw Immunon, Pittsburgh, PA, U.S.A.) and cells were observed under a Zeiss fluorescence microscope equipped with a camera (Zeiss, Thornwood, NY, U.S.A.).

### Flow cytometric analysis

Cells were stained as described above. The mean fluorescence of 10,000 events of nile red stained cells was measured in the FL2 channel set for log scale, while the percentage of ADRP-stained cells was measured in the FL1 channel set for log scale, using FACScan flow cytometry (Becton Dickinson, Italy).

### Lactate dehydrogenase

Lactate dehydrogenase (LDH) leakage, as indicator of cell viability and membrane integrity, was determined in the culture media using a commercially available kit (Sigma). Results are expressed in U/l.

### Reverse Transcriptase-Polymerase Chain Reaction (RT-PCR)

For determination of ADRP mRNA levels, semiquantitative RT-PCR was performed as previously described (Corsini et al. 1999). Briefly, for time course experiments confluent cells cultured in 6 wells plate were treated with SDS (15 μg/ml) in 1.4 ml of media supplemented with 1% FCS for different time points (1–18 h), while to investigate the role of SP-1 in ADRP mRNA expression cells were treated for 1 h with increasing concentrations of mithramycin (0.01–1 μM) or DMSO as vehicle control and, then in the presence or absence of SDS (15 μg/ml) for 6 h. Monolayers were washed once with PBS and cells lysed in 1 ml of Trizol^™^ (Invitrogen, Carlsbad, CA, U.S.A.). Total RNA was extracted following supplier’s instructions. PCR primers for ADRP and glyceraldheyde-6-phosphate dehydrogenase (GPDH) were synthesized and contained the following sequences:

- ADRP: sense, 5′-AGCTGTTGGTAGAACAGTAC-3′
- ADRP antisense, 5′-AGATGTCGCCTGCCATCACC-3′
- GPDH: sense, 5′-CCACCCATGGCAAATTCCATGGCA-3′
- GPDH antisense, 5′-TCTAGACGGCAGGTCAGGTCCACC-3′

The amplified PCR products are 510 bp for ADRP and 600 bp for GPDH. In preliminary experiments, RNA concentrations and PCR cycles were titrated to establish curves to document linearity and to permit semiquantitative analysis of signal strength (1 ng for ADRP and 4 ng for GPDH). The PCR was conducted using a Perkin Elmer thermocycler, with the following conditions: 28 cycles of denaturation at 95 °C for 30 sec, annealing at 60 °C for 30 sec, extension at 72 °C for 30 sec, followed by a final 7 min extension at 72 °C. Gels were photographed with type 55 film (Polaroid, Cambridge, MA, U.S.A.).

### Determination of cytosolic free Ca^2+^ concentration

Cells grown on a glass coverslip were treated in the presence or absence of BAPTA (30 μM) for 30 min, washed and incubated for 3 h with SDS (25 μg/ml). During the last 30 min of incubation, cells were loaded with 10 μM FURA2-AM (Sigma). At the end of the incubation, cells were washed twice and cytosolic calcium was measured in HBSS by the intensity of the fluorescence emission at 505 nm upon excitation at 340–380 nm in a Perkin Elmer LS 50 B double wavelength spectrofluorimeter. The FURA2 fluorescence signal was calibrated in terms of [Ca^2+^]_i_ as described by Grynkiewicz et al. (1985).

### Statistical analysis

All experiments were performed at least three times, with representative results shown. The data presented are expressed as mean ± SD. Statistical significance was determined by Dunnett’s multiple comparison test after ANOVA. Differences were considered significant if p < 0.05.

## Results

### Role of SP-1 in SDS-induced ADRP expression and lipid droplets accumulation

**Figure 1:**
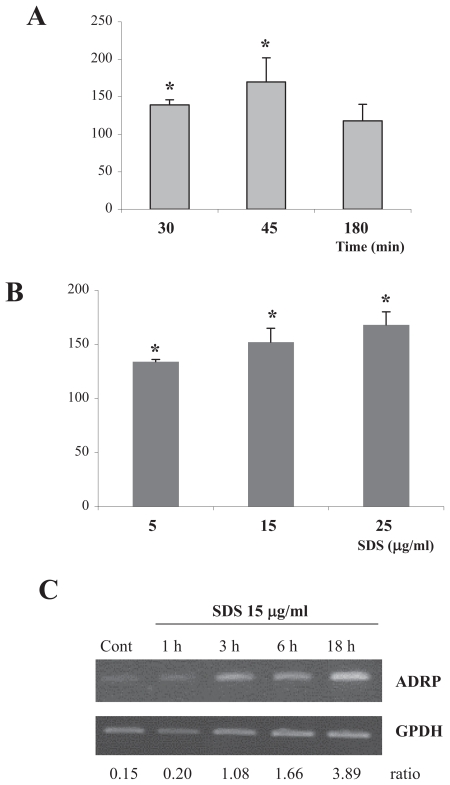
SDS induces a dose and time related activation of SP-1. SDS-induced SP-1 activation is consistent with SDS-induced ADRP mRNA expression. SP-1 activation was assessed by the colorimetric assay described in the Materials and Methods section. **A**) Time course. Confluent cells were treated for different time points (30–180 min) in the presence or absence (control) of SDS 15 μg/ml. **B**) Dose response. Confluent cells were treated with increasing concentrations of SDS (0–25 μg/ml) for 45 min. Results are expressed as % of SP-1 activation relative to control (untreated cells). Each value represents the mean ± SD of three independent samples. *p < 0.05 vs control cells. **C**) ADRP mRNA expression. Confluent cells were treated with SDS 15 μg/ml for different times (0–18 h). mRNA expression was evaluated by semiquantitative RT-PCR.

In order to understand the role of transcription factor SP-1 in SDS-induced ADRP expression, we first assessed the ability of SDS to induce SP-1 activation. As shown in Figure 1, SDS induced a time (1A) and dose related (1B) SP-1 activation, which preceded mRNA accumulation (1C). SDS-induced SP-1 activation reached a maximum after 45 min, and returned to control values after 180 min (Fig. 1A), while SDS-induced ADRP mRNA expression started after 1 h of treatment and increased thereafter (Fig. 1C).

### Mithramycin prevents SDS-induced ADRP expression and lipid droplets accumulation

**Figure 2:**
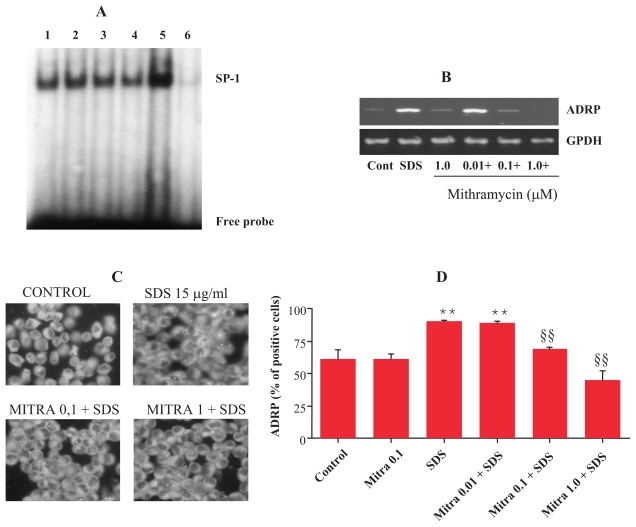
Inhibition of SP-1 by mithramycin prevents SDS-induced ADRP expression and lipid droplets accumulation. Confluent cells were treated with mithramycin (Mitra, 0.01–1 μM) for 1 h and then, in the presence or absence of SDS (15 μg/ml, SDS or +) for different time points. **A**) SP-1 activation evaluated by EMSA. Confluent cells were treated with mithramycin 0.1 μM or DMSO as vehicle control for 1 h and then SDS 15 μ/ml was added for 45 min. Lane 1, control; lane 2, SDS; lane 3, mithramycin; lane 4 mithramycin plus SDS; lane 5, SDS; lane 6, SDS plus cold probe. 5 μg of nuclear extract was used, except for competition for which 10 μg of nuclear extract was used. The migration position of SP-1 protein-DNA complex and of the unbound probe are indicated. **B**) ADRP mRNA expression by RT-PCR analysis. Confluent cells were treated with increasing concentrations of mithramycin 0.01–1 μM or DMSO as vehicle control for 1 h and then SDS 15 μ/ml (+) was added for 3 h. mRNA expression was evaluated by semi-quantitative RT-PCR. **C**) Immunofluorescent localization of ADRP. Cells were treated in the presence or absence of mithramycin 0.1 μM for 1 h and then +/− SDS for 18 h. Cells were trypsinized, fixed and lysed using Leucoperm^™^ followed by incubation with anti human ADRP antibody FITC conjugated for 30 min at RT. Cells were observed under a fluorescence microscope equipped with a camera. **D**) Flow cytometric analysis of ADRP positive cells. Results are expressed as % of positive cells. Each value represents the mean ± SD of 3 independent samples. **p < 0.01 vs control cells, §§p < 0.01 vs SDS treated cells.

To further evaluate the role of SP-1 activation in ADRP expression and lipid droplets accumulation, we examined whether mithramycin A, an antibiotic that selectively binds to GC box preventing SP-1 binding and subsequent gene expression (Ray et al. 1989), could inhibit SDS-induced ADRP expression. As evaluated by EMSA (Fig. 2A) and by a colorimetric assay (Table 1), treatment with mithramycin A prevented in a dose-related manner SDS-induced SP-1 activation. Mithramycin also prevented ADRP mRNA expression (Fig. 2B), as evaluated by semiquantitative RT-PCR, and reduced lipid droplets accumulation (Figs. 2C and 2D), as evaluated by ADPR immunohistochemistry and FACS analysis of ADRP positive cells. The inhibitory effect of mithramycin A was not due to cytotoxicity, as assessed by measuring LDH leakage in culture medium, since no cell death was observed in cells treated with mithramycin 1 μM for up to 18 h (8 ± 5 mU/ml in control vs 6 ± 2 mU/ml in mithramycin treated cells).

### Role of calcium SDS-induced SP-1 activation and lipid droplets accumulation

**Figure 3:**
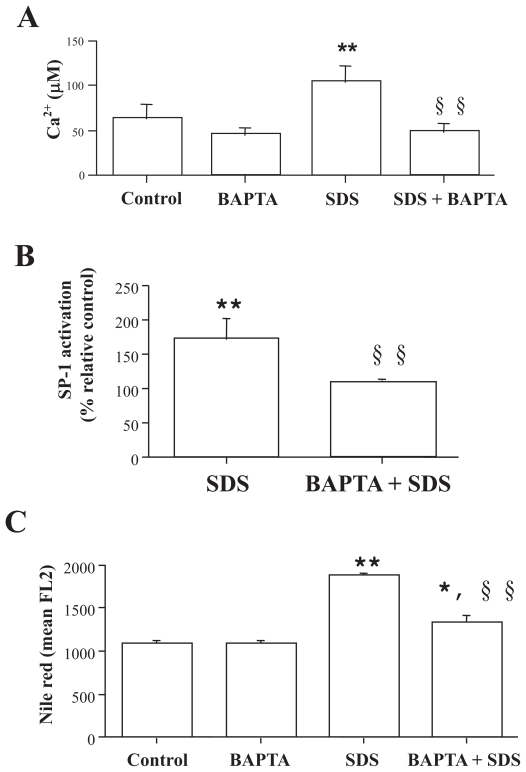
Role of calcium in SDS-induced SP-1 activation and lipid droplets accumulation. Confluent cells were treated with BAPTA 30 μM for 30 min, culture medium was removed, cells washed and then, in the presence or absence of SDS (15 μg/ml) for different times. **A**) Effect of BAPTA on SDS-induced calcium rise. Calcium was measured as described in Materials and Methods section after 3 h of SDS treatment. Each value represents the mean ± SD of three independent experiments. **B**) Effect of BAPTA on SDS (15 μg/ml)-induced SP-1 activation, t = 45 min. SP-1 activation was assessed by the colorimetric assay described in the Materials and Methods section. Results are expressed as % of SP-1 activation relative to control. **C**) Effect of BAPTA on SDS (15 μg/ml)-induced lipid droplets accumulation after 18 h of treatment. Lipid droplets accumulation was assessed by FACS analysis of nile red stained cells. Results are expressed as nile red mean fluorescence (mean FL2). *p < 0.05, **p < 0.01 vs relative control cells; §§p < 0.01 vs SDS-treated cells.

Under experimental conditions, where SDS-induced intracellular calcium increase (Fig. 3A) was completely prevented by using the intracellular calcium chelator BAPTA (from a 171 ± 14% increase to 90 ± 23%), SDS failed to induce SP-1 activation (Fig. 3B), demonstrating a role of calcium in SP-1 activation. SDS alone induced a 170 ± 32% increase in SP-1 activation relative to control values and the treatment with BAPTA completely abolished it (107 ± 3%). Also lipid accumulation, as assessed by nile red staining and FACS analysis (Fig. 3C), was completely prevented by BAPTA.

## Discussion

The purpose of this study was to characterize the role of transcription factor SP-1 in SDS-induced ADRP expression in human keratinocytes. We could show that SDS induces a dose and time related SP-1 activation and that mithramycin A, a selective SP-1 inhibitor, prevents SDS-induced SP-1 activation, ADRP mRNA expression, and lipid droplets accumulation.

We focused our attention on SP-1, since a consensus sequence for this transcription factor, close to the start site (−45), was found in the 5′-flanking region of mouse ADRP gene (Eisinger and Serrero, 1993). Similarly, also in the 5′-flanking region of exon 1 of the human ADRP gene two potential binding sites for the transcription factor SP-1 close to the start site (in position −42 and −58) can been identified. We could demonstrate that the activation of SP-1 plays a crucial role in SDS-induced human ADRP expression.

SP family plays a major role in the expression of numerous housekeeping genes, and SP-1, a member of this family, is an abundant nuclear protein in many cells (Saffer et al. 1991). It has been shown that SP-1 is involved in the expression of cell cycle regulated genes (Karlseder et al. 1996). The role of SP-1 in ADRP expression might also be relevant in cell proliferation, due the important role of ADRP in the management of neutral lipid stores, which are essential cytosolic organelles, used mainly as energy source and for membrane biogenesis.

Overall, little is known about the promoter elements, that regulate the transcription of human ADRP. A peroxisome proliferators-activated receptor (PPAR) response element has been recently identified within the promoter region of the human ADRP gene, that mediates the upregulation of transcription in response to agonists of PPAR in rat and human hepatocyte-derived cell lines (Targett-Adams et al. 2005). Consistently with this finding, it has been demonstrated in keratinocytes that PPAR-beta/delta activation, aside from stimulating keratinocyte differentiation-related genes, improving barrier homeostasis and stimulating triglycerides accumulation induces ADRP (Schmuth et al. 2004). Thus, it is possible that SDS-induced membrane damage may liberate endogenous lipid metabolites, which in turn may activate PPAR or, alternatively, SDS may induce PPAR- beta/delta expression. In addition to PPARs, in macrophages it has been demonstrated that ADRP gene transcription in response to TPA is conjointly regulated by transcription factors PU.1 and AP-1 (Wei et al. 2005).

In the context of skin irritation, we have previously demonstrated that ADRP expression is induced to repair membrane cell damage induced by irritants (Corsini et al. 2003). The surfactant effect of SDS damages lipid bilayers of cell membranes, rendering them leaky and allowing the entrance of extracellular calcium, an event that can trigger the upstream mechanisms that initiate the signaling cascade, result in the activation of SP-1 and culminates with the enhanced ADRP expression. In agreement with the scenario described above, we could demonstrate that SDS-induced SP-1 activation depends on intracellular calcium increase.

Overall, our findings offer insight into the mechanisms, that serve to regulate ADRP expression and demonstrate that SP-1 site is an important cis-acting element regulating its expression.

## Figures and Tables

**Table 1:** Effect of mithramycin on SDS-induced SP-1 activation.

Treatment	SP-1 activation (% of relative control)
SDS 15 μg/ml	139.0 ± 5.3
Mithramycin 10 nM + SDS	120 ± 5.5
Mithramycin 100 nM + SDS	98.0 ± 16.0*
Mithramycin 1000 nM + SDS	99.8 ± 8.6*

Confluent cells were treated with mithramycin (10–1000 nM) for 1 h and then, in the presence or absence of SDS (15 μg/ml, SDS) for 45 min. SP-1 activation was assessed by a colorimetric assay. Results are expressed as % of SP-1 activation relative to control. Each value represents the mean ± SD of three independent samples.

* p < 0.05 vs SDS treated cells.

## List of abbreviations

ADRP: adipose differentiation related protein
BAPTA: 1,2-bis(2-aminophenoxyethane-N,N,N′,N′-tetraacetic acid acetoxymethyl ester
DMSO: dimethyl sulfoxide
DTT: dithiothreitol
EDTA: ethylenediaminetetraacetic acid disodium salt
2-AM: fura-2 pentakis(acetoxymethyl) ester
EGR1: early growth response-1
EMSA: electrophoretic mobility shift assay
FL: fluorescence
GPDH: glyceraldheyde-6-phosphate dehydrogenase
IL: interleukin
LDH: lactate dehydrogenase
PBS: phosphate buffered saline
PPAR: peroxisome proliferators-activated receptor
RT-PCR: reverse transcriptase-polymerase chain reaction
SDS: sodium dodecyl sulfate
TBE: tris borate EDTA
TPA: 12-O-tetradecanoylphorbol 13-acetate
TRE: thyroid hormone receptor
WT1: Wilms tumor locus genes
